# Cognitive Behavior Therapy for Depersonalization-Derealization Disorder (CBT-f-DDD): a feasibility randomized trial

**DOI:** 10.1186/s40814-025-01742-1

**Published:** 2025-12-10

**Authors:** Elaine C. M. Hunter, Lucy Ring, Rafael Gafoor, Nicola Morant, Glyn Lewis, Joe Perkins, Nicola Dalrymple, Ana Dumitru, Cheuk Lon Malcolm Wong, Elena Pizzo, Georgia McRedmond, Anthony S. David

**Affiliations:** 1 Division of Psychiatry, University College London, London, UK; 2 Comprehensive Clinical Trials Unit, Institute of Clinical Trials and Methodology, University College London, London, UK; 3 Unreal Charity, Bristol, UK; 4 Royal Holloway, University of London, Egham, Surrey UK; 5 Department of Primary Care and Population Health, University College London, London, UK

**Keywords:** CBT, Depersonalization, Derealization, Feasibility, RCT

## Abstract

**Background:**

Depersonalization-derealization disorder (DDD) is characterized by feelings of “unreality” about the self and/or external world. Cognitive Behavioral Therapy adapted for DDD (CBT-f-DDD) has been effective in published clinical audits. This study aimed to provide feasibility and acceptability data.

**Methods:**

An individually randomized design of CBT-f-DDD versus Treatment as Usual (TAU) was carried out with adult DDD participants from NHS Trusts in London. The CBT-f-DDD group received individual sessions over a 6-month period from CBT therapists. Qualitative interviews were conducted with CBT-f-DDD participants and their clinicians. Eight feasibility objectives were evaluated (recruitment, retention, resources, representativeness, acceptability of study design and intervention, preliminary responses to intervention, and health economics).

**Results:**

Thirty participants with DDD were recruited over 13 months. Only 63% completed the final assessment, so retention needs improvement. Resources were acceptable. The sample was comparable to previous studies, although younger, with a shorter duration of DDD and lower mean DDD scores. In a post-study questionnaire, no aspect of the study or treatment was rated unacceptable; however, some areas need improvement. Qualitative interviews with participants and clinicians recorded positive responses to CBT-f-DDD. Those in the CBT arm had a mean decrease of 16.9 points (SD 43.6) on the Cambridge Depersonalization Scale versus a mean decrease of 5.5 points (SD = 25.0) for the TAU arm. Health economics analyses found that CBT-f-DDD saved £153 per person. Participants reported an additional 0.08 Quality-Adjusted Life Years at low cost.

**Conclusions:**

This study suggests that a subsequent RCT for CBT-f-DDD is feasible and represents the first step in the process of establishing evidence-based treatments for DDD. However, refinements to the current design and delivery were indicated for a future fully powered definitive RCT of CBT-f-DDD.

**Trial registration:**

ISRCTN, ISRCTN97686121. Retrospectively registered 5 January 2023: https://doi.org/10.1186/ISRCTN97686121

## Key messages regarding feasibility

1. Uncertainties regarding feasibility included whether sufficient participants with DDD could be recruited from NHS services; whether these participants could be retained for assessment and treatment; what clinical and research resources would be needed; whether participants with DDD in general NHS services would be similar to those evaluated in previous studies from specialist services; whether the study processes, measures and intervention would be acceptable to participants and clinicians; whether generic CBT therapists could be trained to deliver CBT-f-DDD; whether the preliminary evaluation of the intervention appeared promising; was there any evidence suggesting superior efficacy of the CBT treatment versus TAU; and would there be health economic benefits to CBT-f-DDD.
2. The key feasibility findings were that DDD recruitment was possible, especially within primary care Talking Therapy services, albeit slow; retention was lower than hoped for (63%), however comparable to local services. Resources required were acceptable. The sample recruited for this study were comparable to previous samples although younger, with shorter and less severe DDD, but with more comorbidities. The acceptability of data collection was good; however, there was more mixed acceptability of study processes and intervention, although none were deemed unacceptable. It was feasible to train clinicians to deliver CBT-f-DDD. Preliminary evaluation of the response to intervention was promising albeit less than in previous studies, perhaps demonstrating that the previous studies had suffered potentially from a combination of biases and unadjusted confounding. The health economics analysis found that CBT-f-DDD had both individual and societal cost benefits.
3. The implications of the feasibility findings for the design of the main study are that although it is possible to recruit participants with DDD, more sites would be needed, in a wider range of services and settings across the UK. Further strategies to increase retention would be indicated such as directly employing clinicians in the study so that participants could continue to be offered treatment if they relocate, having greater continuity of study staff conducting assessments and offering more in-person sessions to the CBT group as those with DDD report virtual sessions can emphasize their sense of disconnection. In addition, having trial therapists working specifically on CBT-f-DDD would allow greater homogeneity of clinical experience levels and less competing demands on their time to improve session delivery continuity and therapists' participant acceptability. Increasing the training period to 1 day and for therapists to have a practice client before starting with trial clients would hopefully increase acceptability ratings from participants. The preliminary results show that there is evidence that CBT is more effective than TAU for the treatment of DDD symptoms, and a fully powered study is indicated so that inferential testing can be conducted on an adequate sample size.

## Background

Depersonalization-Derealization Disorder (DDD) is a distressing mental health condition where a person has persistent symptoms or episodes characterized by a profound sense of disconnection and unreality about themselves (depersonalization) and/or the world (derealization), alongside a spectrum of other symptoms which may include emotional blunting as well as physiological, perceptual and cognitive impairments [1]. Systematic reviews of the prevalence of DDD in community surveys estimate it to be approximately 1% [2, 3]. However, there is a large gap between population prevalence rates and clinical diagnosis due to factors such as a lack of clinician knowledge with pervasive under-diagnosing of DDD undoubtedly contributing to the widely held, erroneous assumption that DDD is rare [4]. Consequently, there has been underfunding of research into effective treatments, and a limited evidence base. There is currently no National Institute for Health and Care Excellence (NICE) guidance on treatments for DDD in the UK.

CBT is the most widespread talking therapy used in the NHS with effectiveness demonstrated across a wide range of conditions [5]. In a systematic review of the existing literature on DDD treatment, improvements in symptoms were found across medication, repetitive transcranial magnetic stimulation and psychological therapies, with CBT studies providing the most robust evidence amongst therapeutic modalities [6]. There have been two studies of audit data from consecutive referrals to a tertiary specialist clinic [7, 8]. In the first of these, where 21 clients received CBT-f-DDD, there was a significant reduction in state DDD symptom severity from pre-treatment to 6-month follow-up (adjusted mean difference of 12.6 (SD = 19.5); mean effect size = 0.37; [95% CI, 0.12 to 0.53]), and 29% of participants no longer met diagnostic criteria for DDD at the end of therapy. A second, recent analysis of outcomes of 36 participants has replicated these initial findings. In a cross-over design which compared each participant’s DDD score while on a waiting list versus their score once they received CBT therapy, significant improvements were found on trait DDD symptoms (adjusted mean difference of −35.99 points on the CDS scale; [95% CI, −48.45 to −23.52]); mean effect size of − 0.52 [− 0.70 to − 0.34]) after a mean of 18 sessions of CBT, along with reductions in anxiety and depression symptoms.

However, there were several methodological limitations of both these studies including the following: use of non-randomized audit data; small sample sizes; participants only recruited from one tertiary specialist clinic (and therefore perhaps not representative of the larger at-risk population); and a small number of clinicians who delivered the intervention. It is not possible, therefore, to know for certain if the response to CBT-f-DDD was affected by unadjusted confounding bias and whether these results can be generalized to other NHS settings. However, given the chronic nature of DDD, these results are still promising.

Given the gap in empirical research, the present study aimed to undertake a feasibility Randomized Controlled Trial (RCT) of CBT-f-DDD. There were 8 objectives overall, which included those outlined as essential for a feasibility study [9], namely the evaluation of:

1. Recruitment
2. Retention
3. Resources needed
4. Representativeness
5. Acceptability of data collection procedures and measures
6. Acceptability of intervention
7. Preliminary evaluation of the magnitude and direction of the difference in outcome scores between the two treatment arms
8. Health economics analysis

## Methods

This section provides a summary of the trial protocol [10].

### Design

A two-arm parallel group (blinded to assessor and statistician) individually randomized design, consisting of CBT-f-DDD versus Treatment as usual (TAU). Consented participants were randomized by sealed envelope method, with randomization lists created by an independent statistician prior to recruitment.

### Participants

Inclusion criteria were that participants were aged between 16 and 75 and met current DSM-V criteria for DDD. Exclusion criteria included having previously received CBT-f-DDD, inability to consent, insufficient proficiency in English, not GP registered, living out of the catchment area of the treatment centers participating in the study, involvement in other research, having a diagnosis of psychosis, substance dependence, or cognitive impairment due to head injury/organic disorder. Those with a diagnosis of current PTSD were also excluded, as a trauma-focused treatment protocol, rather than CBT-f-DDD, would be indicated in these cases.

As this was a feasibility study, there was no formal sample size calculation or a priori power calculation. To encompass a nominal 20% dropout rate to ensure the recommended minimum number for a feasibility study in each treatment arm of 12 participants [11], we aimed to recruit 30 participants overall to give a sample large and diverse enough to assess the practicalities of recruitment, retention, randomization, and intervention delivery, and to obtain the necessary data for the statistical inferences required.

### Procedure

Participants were recruited between May 2022 and May 2023 via three London NHS mental health Trusts, across primary, secondary, and tertiary services. Potential participants were identified via clinicians as well as through self-identification of those from the UK DDD charity *Unreal* website who were resident in the Trusts taking part. Potential participants were provided with a Participant Information Sheet, and if written consent was given, they completed an eligibility screening. Those recruited completed assessments at four timepoints: baseline (T0); one month after baseline (T1) at which point participants had been assigned to a therapist and were starting therapy; six months after baseline (T2); and nine months after baseline (T3). Participants completed the Cambridge Depersonalization Scale (CDS), standardized clinical and economic measures, and a semi-structured interview with a research assistant at T0, T2, and T3. Participants completed self-report measures on their own at T1. Upon T3 completion, all participants completed a study satisfaction questionnaire. Participants and clinicians in the CBT condition were invited to take part in an additional qualitative interview. Upon completion of all assessments, participants in both groups received a £10 voucher.

Clinicians were recruited from a range of services within the three participating NHS Trusts. They attended a half-day training workshop on CBT-f-DDD, were supplied with a manualized protocol, and if allocated a participant, attended fortnightly clinical supervision.

PPI involvement was embedded throughout the study, with *Unreal* who was a co-applicant in the study. Eight PPI members with lived experience of DDD were recruited through Unreal to a Patient Advisory Group (PAG) who met at critical points in the study process. Three PAG meetings were held between March 2022 and June 2024 at key stages of the study (namely, design, recruitment and results) and suggested changes were made. A representative of Unreal attended monthly Trial Management Group meetings, created videos for recruitment and dissemination of the results, trained research assistants, and contributed to qualitative data analysis.

### Intervention

Participants randomized to the CBT-f-DDD arm were offered individual sessions during a 6-month intervention window, delivered by an NHS therapist working within their own service who had received training in the intervention. The clinician attended fortnightly specialist group supervision (led by ECMH) while providing the intervention. CBT-f-DDD aims to reduce distress associated with DDD symptoms through psychoeducation about dissociative processes, developing an individualized shared formulation of their DDD with maintaining factors, and working to instigate cognitive restructuring and behavioral change. Table 1 shows the key interventions and stages of CBT-f-DDD. Those in the TAU condition were asked to note whatever interventions were offered to them during the 6-month intervention window (including therapy, medication review, general support, or waiting list). These data were supplemented with information from clinical notes.

**Table 1:** CBT-f-DDD treatment stages

**Engagement and psychoeducation about DDD**
• Managing expectations and goal setting
• General and individualized psychoeducation about DDD
**Developing the shared formulation**
• Diary keeping and analysis
• Identifying external and/or internal triggers
• CBT vicious cycle of recent, typical incident
**Cognitive strategies: content and process**
• Restructuring beliefs about DDD
• Recognizing thinking biases
• Working with cognitive processes to reduce rumination, worry and hypervigilance
**Emotional regulation strategies**
• Anxiety and mood management strategies
• Grounding strategies
**Behavioral Interventions**
• Psychoeducation about safety seeking behaviors
• Behavioral activation
• Behavioral experiments
• Graded exposure
**Working with common comorbid conditions triggering DDD**
• Anxiety disorders, low mood, low self-esteem
• Working with negative core beliefs/schemas, procrastination, perfectionism
• Substance use
**Working with issues related to onset**
• Trauma-focused CBT imaginal exposure
• Dealing with physical reminders of onset
• Chair work where the client uses different chairs to represent different aspects of themselves to foster communication and insights from taking various perspectives
**Working with predisposing factors**
• Working with past trauma or life adversity
**Staying well plans**

### Data collection and measures

Feasibility of trial recruitment (Objective 1) was assessed by monitoring numbers and rates of referrals from each source, number of ineligible referrals, reasons for ineligibility, as well as rates and reasons for refusal to take part in the study. Retention (Objective 2) was assessed by rates of attrition and reasons for withdrawing from the study. Resources needed (Objective 3) were assessed by calculating the time of both research and clinical staff to complete the study.

Measures for representativeness of the sample (Objective 4) included questions regarding demographic, DDD, and other relevant clinical data, collected at baseline and follow-up time points. A questionnaire designed specifically for the study asked about DDD history, including age of DDD onset, duration, current/past patterns of DDD symptoms, and current/past treatments for DDD. The following standardized measures were used (see published trial protocol [10] for full description):

1. Cambridge Depersonalization Scale (CDS; [12])
2. Dissociative Experiences Scale-II (DES-II; [13])
3. Patient Health Questionnaire-9 (PHQ-9; [14])
4. Generalized Anxiety Disorder Assessment-7 (GAD-7; [15])
5. Clinical Interview Schedule-Revised (CIS-R; [16, 17])
6. Work and Social Adjustment Scale (WSAS; [18])

Participants’ acceptability of data collection measures and processes (Objective 5) and the intervention (Objective 6) were assessed using both quantitative and qualitative methods. A satisfaction questionnaire designed specifically for the study was administered at T2 for all participants. This questionnaire contained six questions to assess acceptability of the study (How helpful was the information you were given about the study and treatment?; How satisfied were you with the randomization process?; How would you rate the assessment process?; How would you rate the questionnaires you were given?; How satisfied were you with how your queries were dealt with?; How would you rate your overall experience with this study?). Four further questions assessed acceptability of treatment (How satisfied were you with the therapist you have worked with? How would you rate the outcome of the treatment you have received? How would you rate your symptoms of DDD after having received treatment? How satisfied were you with the treatment you have received?). Qualitative semi-structured interviews were conducted after T3 with a sub-sample of participants who received the intervention. Therapists’ acceptability of data collection and intervention were assessed with semi-structured qualitative interviews after they had completed therapy with all clients.

To measure the preliminary evaluation of efficacy (Objective 7), the magnitude and direction of the difference between scores for the CDS scale between the two arms was used. A simple global rating of change question (i.e., “do you feel better?”) was also included.

Health economics data were collected at T0, T2, and T3. Quality of life of patients was assessed using the EuroQol EQ5D3L questionnaire [19], as per NICE guidelines. A modified version of the Client Service Receipt Inventory (CSRI; [20]) was administered to collect patient-level data on NHS and private healthcare consumption (GP consultations, inpatient, outpatient and community services; medications) and data on productivity losses (days off work).

Any incidents of harm or unintended effects in either arm of the study were logged during the study.

A list of the prespecified a priori progression criteria that needed to be met to establish the feasibility of the full trial is provided in Table 2. Red/Amber/Green (RAG) criteria were used to monitor progress on the outcomes of the study throughout the trial. The trial was registered: ISRCTN (10.1186/ISRCTN97686121).

**Table 2:** RAG feasibility criteria

Feasibility Criteria	Results
**Recruitment/eligibility data** Number of referrals from each source, numbers ineligible, reasons for ineligibility, rates and reasons for refusal to be included in the study. A minimum of 12 participants in each arm for feasibility studies has been suggested for pilot studies which would require recruiting 29 eligible participants in total (including 20% drop out estimate) During a 6-month window, to reach the recruitment target of 29–60 participants, we would need to recruit at a rate of 8–10 participants per month, or 1–2 per week • Green: at least 2 participants per week, or at least 8 per month • Amber: 1–2 participants per week, or 5–8 per month • Red: less than 1 participant per week, less than 5 per month	Red
**Participant attrition rates and reasons for withdrawing throughout the study, follow-up rates** Design allows for a 20% drop-out rate • Green: up to 20 participants drop out • Amber: up to 30 participants drop out • Red: more than 30 participants drop out	Red (38% drop out across both groups)
**Therapist attrition rates and reasons for withdrawing throughout the study** • Green: 10–20 therapists (to completion) • Amber: 10 therapists (to completion) • Red: < 10 therapists (to completion)	Green
**Resources needed to complete CBT-f-DDD** A minimum of 12 sessions and maximum of 24 weekly sessions are established for the 6-month window, allowing for 50% attendance rates • Green: over 50% sessions attended (*n*=12+) • Amber: 50% sessions attended (*n*=12) • Red: less than 50% sessions attended (*n*< 12)	Green
**Therapist adherence to CBT-f-DDD (fidelity)** A random sample of 10% of audio-recorded therapy sessions will be evaluated using standardized CBT protocols for adherence to the CBT model as well as an adapted CBT-f-DDD checklist • Green: Cognitive Therapy Scale—Revised mean score of > 3 (total score 36–72), i.e. proficient range • Amber: Cognitive Therapy Scale—Revised mean score of = 3 (total score 36), i.e. competent range • Red: Cognitive Therapy Scale—Revised mean score of < 3 (total score less than 36), i.e. incompetent/novice/advanced beginner range	Between Green and Amber
**Participant acceptability** Acceptability of CBT-f-DDD including aspects of the treatment found helpful and unhelpful, perceived impact of the intervention, and satisfaction with the intervention and therapists (from thematic analysis of the qualitative interviews; and as measured by items on the end-of-treatment questionnaire “CBT-f-DDD brief outcomes and experiences questionnaire”)	
1. How helpful was the information you were given about the study and treatment? • Green: > 50% responding somewhat/extremely helpful • Amber: > 50% responding neither helpful nor unhelpful • Red: > 50% responding somewhat/extremely unhelpful	Between green and amber
2. How satisfied were you with the randomization process (i.e., having a 50/50 chance to be allocated to the CBT-f-DDD group or standard care group)? • Green: > 50% responding somewhat/extremely satisfied • Amber: > 50% responding neither satisfied nor dissatisfied • Red: > 50% responding somewhat/extremely dissatisfied	Between green and amber
3. How would you rate the assessment process? • Green: > 50% responding very good/excellent • Amber: > 50% responding good/fair • Red: > 50% responding poor/very poor	Green
4. How would you rate the questionnaires you were given? • Green: > 50% responding very good/excellent • Amber: > 50% responding good/fair • Red: > 50% responding poor/very poor	Green
5. How satisfied were you with how your queries were dealt with? • Green: > 50% responding somewhat/extremely helpful • Amber: > 50% responding neither helpful nor unhelpful • Red: > 50% responding somewhat/extremely unhelpful	Green
6. How satisfied were you with the therapist you have worked with? • Green: > 50% responding somewhat/extremely helpful • Amber: > 50% responding neither helpful nor unhelpful • Red: > 50% responding somewhat/extremely unhelpful	Between Green to Amber
7. How would you rate the outcome of the treatment you have received? • Green: > 50% responding very good/excellent • Amber: > 50% responding good/fair • Red: > 50% responding poor/very poor	Between Green to Amber
8. How would you rate your symptoms of Depersonalization/Derealization after having received treatment? • Green: > 50% responding a little better/much better • Amber: > 50% responding the same • Red: > 50% responding a little worse/much worse	Between Green to Amber
9. How satisfied were you with the treatment you have received? • Green: > 50% responding somewhat/extremely helpful • Amber: > 50% responding neither helpful nor unhelpful • Red: > 50% responding somewhat/extremely unhelpful	Between Green to Amber
10. How would you rate your overall experience with this study? • Green: > 50% responding very good/excellent • Amber: > 50% responding good/fair • Red: > 50% responding poor/very poor	Between Green to Amber
**Therapist acceptability** Satisfaction with training and supervision, confidence in delivering CBT-DDD, views of the intervention’s value and perceived impact, and perceived feasibility of delivery in standard clinical settings (measured by workshop feedback training satisfaction overall rating score and thematic analysis of the qualitative interviews): • Green: training satisfaction score of 6–8 • Amber: training satisfaction score of 3–5 • Red: training satisfaction score of 0–2	Green

### Analysis

Descriptive analyses were performed on all outcomes. The pooled standard deviation for the baseline score of the CDS was determined, as well as the correlation of the CDS at baseline and nine-month follow-up. No inferential testing was performed; however, the magnitude and direction of the difference between the outcome scores for the two arms was interpreted as evidence of efficacy (or not) of the intervention. Qualitative data from semi-structured interviews were transcribed and analyzed using codebook thematic analysis within Nvivo software. This was guided by Braun & Clarke’s [21] and King’s six-stage process [22].

## Results

### Objectives 1: Recruitment

#### Participants

**Fig. 1:**
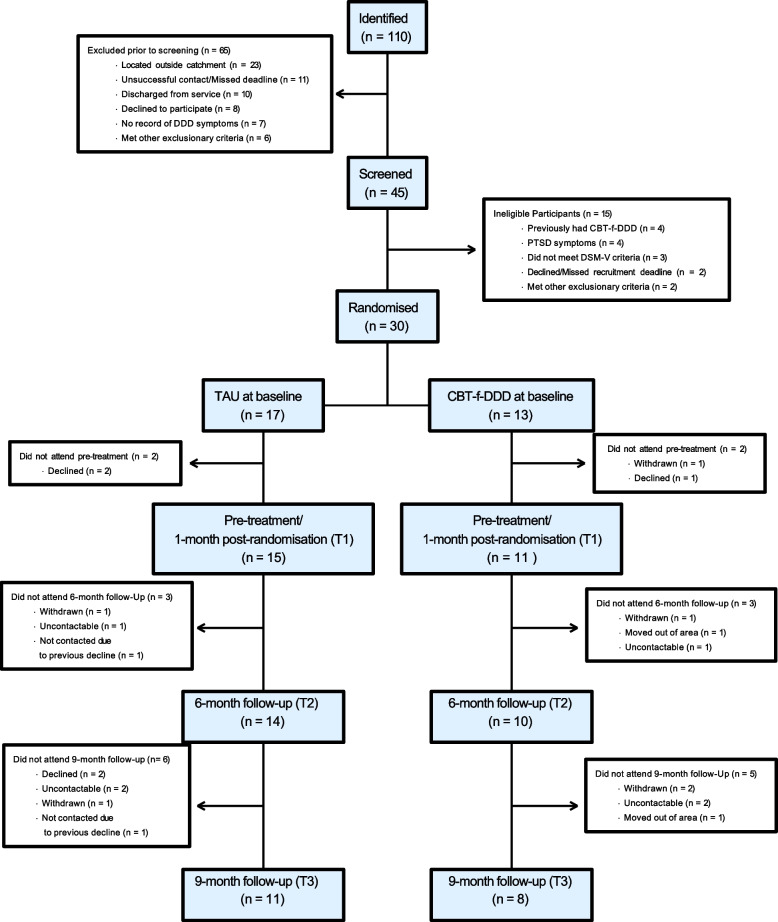
Consort diagram

Thirty participants were eligible for inclusion and consented to participate and were randomized to receive CBT-f-DDD (*N* = 13; 43%) or TAU (*N* = 17; 57%) (see Consort diagram [Fig. 1]).

In terms of distribution across Trusts, of the 30 who took part, 90% (*n* = 27) were from Camden and Islington NHS Foundation Trust (C&I), 7% (*n* = 2) were from Barnet, Enfield and Haringey Mental Health NHS Trust (BEH) and 3% (*n* = 1) was from Whittington Health Trust (WH). Participants were mainly from primary care Talking Therapies services (73% (*n* = 22)), 17% (*n* = 5) were from general primary mental health care services, and 10% (*n* = 3) from secondary care services.

Participant recruitment rates were lower than had been estimated at an average of 2.3 per month. This was largely due to two reasons: less engagement from some of the NHS services involved, and because many potential participants who self-referred via the charity Unreal were outside the London Trust catchment area. Recruitment was RAG (Red-Amber-Green) rated as red during the study (Table 2), and remedial actions were taken during the study to allow it to continue to meet the minimum number of participants needed. These included finding additional sources of recruitment and requesting Clinical Research Network assistance. In addition, the study design was altered to allow for a longer recruitment period by decreasing the follow-up period from the original plan of 6 months after T2 to 3 months after T2, so the study could be completed within the agreed timeframe and budget.

#### Therapist recruitment

Thirty-seven NHS clinicians attended half-day training workshops between March 2022 and April 2023. There were five workshops run in total. Seven clinicians took at least one participant for CBT-f-DDD. All seven worked in Talking Therapies services, and the median time since qualifying was 7 years (IQR, 8.25).

### Objective 2: Retention rates

#### Withdrawals

Three participants formally withdrew from the study (two in the CBT arm and one in the TAU arm). In the CBT group, one participant withdrew at T1 without a reason, and the second participant withdrew at T3 because they moved out of the area and so could not receive treatment from the participating NHS services. One participant in the TAU group withdrew at T2, giving no reason.

#### Assessment of retention rates

The percentage of participants who completed the outcome measure (CDS) was T1 = 87% (*n* = 26); T2 = 80% (*n* = 24); T3 = 63% (*n* = 19). The overall dropout rate for T3 assessment at 37% for participants in the CBT group was red RAG rated (Table 2). There was similar attrition between the two arms of the study. The main reason for attrition for assessment was participant relocation outside of the catchment area of the study so they could not be offered treatment, and although numerous attempts were made to maintain contact, they were lost to follow up.

#### Therapy retention rates

The mean number of CBT-f-DDD sessions was 13 (SD = 7). We had set an a priori minimum “dose” of therapy for minimum clinical effectiveness as being 6 sessions, as this had been used in a previous study [8]. Of the 13 participants allocated to CBT-f-DDD, 54% (*n* = 7) completed ≥ 6 sessions, 23% (*n* = 3) completed 2–5 sessions, and 23% (*n* = 3) completed no sessions. The rates of retention in therapy for those who attended ≥ 6 sessions were similar in both CBT-f-DDD (54%) and TAU (53%) groups. The early dropout rates for CBT-f-DDD of 23% were comparable with CBT for other conditions in a recent Talking Therapies clinical audit for the same C&I service (personal communication) where 21% dropped out after two sessions. Therapy retention rates were RAG rated green.

#### Clinician retention rates

There were no attrition rates for therapists throughout the study (RAG rated green).

### Objective 3:Resources required for CBT-f-DDD

Two hundred twenty-six sessions were attended by both groups (CBT = 127; TAU = 99). In the CBT condition, participants attended more sessions (mean = 12.7, SD = 7.12, range = 2 to 21) than those in TAU (mean = 9, SD = 4.47, range 2 to 16); however, this difference was not statistically significant. The CBT group attendance rate was lower than that in the TAU condition (76% vs. 90%). Therapy was delivered to both groups via videoconferencing (67%), in-person sessions (22%), and phone sessions (1%), with a further 10% of missing data on the modalities employed.

#### Staff resources

A full-time research assistant was employed for the duration of the study, and an estimated 171 hours were required for contacting and meeting with participants, data collection, and entry. Group clinical supervision of up to 4 clinicians was offered by a consultant clinical psychologist (ECMH) on a weekly basis for 90 minutes over the course of the intervention phase (21 months) totaling approximately 120 hours.

#### Treatment fidelity

A random sample of 10% of therapy sessions that had been audio recorded was assessed using the revised Cognitive Therapy Scale (CTS-R; [23]). These sessions were rated in the competent range (amber to green RAG rated).

Therapists rated their clients’ engagement with CBT-f-DDD for nine participants in the CBT condition. In terms of engagement with therapy, 56% (*n* = 5) showed excellent, very good, or good engagement, 22% (*n* = 2) were rated average, and 22% (*n* = 2) were rated as having no/minimal or some engagement. Engagement with CBT-f-DDD homework was mixed with each of the three categories of good–excellent/average/none-some having 33% (*n* = 3) in each category.

### Objective 4: Representativeness

Tables 3, 4, and 5 present, respectively, participant characteristics in terms of baseline demographic data, DDD information, and other clinical characteristics.

**Table 3:** Baseline demographic characteristics

	**Treatment arm**					
	**TAU** *N*** = 17 (57%)**		**CBT** *N*** = 13 (43%)**		**Total** *N*** = 30 (100%)**	
	**Mean**	**(SD)**	**Mean**	**(SD)**	**Mean**	**(SD)**
**Age**	26.53	(5.51)	27.69	(6.26)	27.03	(5.77)
	***n***	**(%)**	***n***	**(%)**	***n***	**(%)**
**Sex**						
Male	6	(35)	6	(46)	12	(40)
Female	11	(65)	7	(54)	18	(60)
**Ethnicity**						
White	12	(71)	12	(92)	24	(80)
Mixed background	3	(18)	0	(0)	3	(10)
Other ethnic group	1	(6)	1	(8)	2	(7)
Missing	1	(6)	0	(0)	1	(3)
**Type of employment**						
Paid or self-employed	12	(71)	6	(46)	18	(60)
Unemployed	4	(24)	2	(15)	6	(20)
Student	1	(6)	5	(38)	6	(20)
**Highest level of education**						
Secondary education	5	(29)	3	(23)	8	(27)
Tertiary/further education	12	(71)	10	(77)	22	(73)

**Table 4:** DDD characteristics at baseline

	**Treatment arm**					
	**TAU ***N*** = 17 (57%)**		**CBT ***N*** = 13 (43%)**		**Total ***N*** = 30 (100%)**	
	**Median**	**(IQR)**	**Median**	**(IQR)**	**Median**	**(IQR)**
**Age chronic DDD started**	17.50	(15.75–23.50)	21.00	(18.80–24.50)	20.00	(16.00–24.50)
**Duration of DDD (years)**	5.00	(1.00–10.25)	4.00	(1.00–7.00)	4.00	(1.00–9.00)
**CDS Score**	123	(86–170)	112	(94–145)	122	(94–170)
**DES-II Total**	27.12	(21.18–39.78)	33.47	(20.36–39.52)	32.60	(20.55–39.78)
**DES-II: DDD Subscale**	31.67	(21.67–50.00)	40.00	(31.67–53.33)	32.50	(28.33–53.33)
**Prior treatment for DDD**	***n***	**(%)**	***n***	**(%)**	***n***	**(%)**
No	10	(59)	9	(69)	19	(63)
Yes	7	(41)	4	(31)	11	(37)

**Table 5:** Other clinical characteristics at baseline

	**Treatment arm**					
	**TAU ***N*** = 17 (57%)**		**CBT ***N*** = 13 (43%)**		**Total ***N*** = 30 (100%)**	
	Median	**(IQR)**	Median	**(IQR)**	Median	**(IQR)**
GAD-7 score	13.00	(10.00)	13.00	(5.00)	13.00	(8.00)
PHQ-9 score	13.00	(8.00)	11.00	(8.00)	12.00	(8.00)
WSAS Score	25.00	(13.00)	22.00	(4.00)	23.50	(10.00)
CIS-R Score	32.00	(22.00)	36.00	(11.00)	34.50	(19.00)
**Number of medications**	***n***	**(%)**	***n***	**(%)**	***n***	**(%)**
0	7	(41)	8	(62)	15	(50)
1	4	(24)	4	(31)	8	(27)
2 +	6	(35)	1	(15)	7	(23)
**Type of psychiatric medication**						
Antidepressants	6	(35)	1	(8)	7	(23)
Anxiolytics	2	(12)	0	(0)	2	(7)
Antipsychotics	2	(12)	0	(0)	2	(7)
**Current co-morbid CISR Diagnosis**						
No diagnosis	3	(18)	1	(8)	4	(13)
Depression: severe	0	(0)	2	(15)	2	(7)
Depression: moderate	3	(18)	5	(38)	8	(27)
Depression: mild	1	(6)	0	(0)	1	(3)
Generalized Anxiety Disorder	5	(29)	4	(31)	9	(30)
Specific phobia	2	(12)	0	(0)	2	(7)
Mixed anxiety and depression	3	(18)	1	(8)	4	(13)

The mean age of the sample was 27.0 years (SD = 5.77). Sixty percent were female. The median age of DDD symptoms becoming chronic was 20 years old, and the median DDD duration was four years. The median CDS score at baseline was 122, where the range of scores is from 0 to 290, and a score of 70 is correlated with a clinical diagnosis [12]. Eleven out of 30 participants (37%) had received some prior treatment for their DDD. This was primarily in the form of medication or some form of psychological therapy, although not specifically CBT for their DDD as this was an exclusion criterion. Median scores at baseline on the GAD-7 and PHQ-9 were 13 and 12 respectively, which indicated anxiety and depression of moderate severity. In addition, the CIS-R indicated probable ICD-10 diagnoses of depression (37%, *n* = 11), Generalized Anxiety Disorder (30%, *n* = 9), and mixed anxiety and depression (13%, *n* = 4).

### Objectives 5 and 6: Participant acceptability of the study and intervention

#### Quantitative analyses

Twenty-two participants (73%) completed the satisfaction with treatment questionnaire at T2 regarding acceptability for both data collection and intervention. Table 6 shows full results by group.

**Table 6:** Participant quantitative satisfaction with study and treatment

	**TAU** (*N* = 17) *n* (%)	**CBT ** (*N* = 13) *n* (%)	**Total ** (*N* = 30) *n* (%)
**How helpful was the information you were given about the study and treatment?**			
Extremely helpful/Somewhat helpful	5 (29)	6 (46)	11 (37)
Neither helpful nor unhelpful/Somewhat unhelpful/Extremely helpful	8 (47)	3 (23)	11 (37)
Unanswered	4 (24)	4 (31)	8 (27)
**How satisfied were you with the randomization process?**			
Extremely satisfied/Somewhat satisfied	3 (18)	5 (38)	8 (27)
Neither satisfied nor dissatisfied/Somewhat dissatisfied/Extremely dissatisfied	10 (59)	4 (31)	14 (47)
Unanswered	4 (24)	4 (31)	8 (27)
**How would you rate the assessment process?**			
Excellent/Very good/Good	8 (47)	9 (69)	17 (57)
Fair/Poor/Very poor	5 (29)	0 (0)	5 (17)
Unanswered	4 (24)	4 (31)	8 (27)
**How would you rate the questionnaires you were given?**			
Excellent/Very good/Good	9 (53)	7 (54)	16 (53)
Fair/Poor/Very poor	4 (24)	2 (15)	6 (20)
Unanswered	4 (24)	4 (31)	8 (27)
**How satisfied were you with how your queries were dealt with?**			
Extremely satisfied/Somewhat satisfied	10 (59)	6 (46)	16 (53)
Neither satisfied nor dissatisfied/Somewhat dissatisfied/Extremely dissatisfied	3 (18)	2 (15)	5 (17)
Not applicable	0 (0)	1 (8)	1 (3)
Unanswered	4 (24)	4 (31)	8 (27)
**How satisfied were you with the therapist you have worked with?**			
Extremely satisfied/Somewhat satisfied	6 (35)	7 (54)	13 (43)
Neither satisfied nor dissatisfied/Somewhat dissatisfied/Extremely dissatisfied	4 (24)	2 (15)	6 (20)
Not applicable	3 (18)	0 (0)	3 (10)
Unanswered	4 (24)	4 (31)	8 (27)
**How would you rate the outcome of the treatment you have received?**			
Excellent/Very good/Good	5 (29)	6 (46)	11 (37)
Fair/Poor/Very poor	4 (24)	3 (23)	7 (23)
Not applicable	4 (24)	0 (0)	4 (13)
Unanswered	4 (24)	4 (31)	8 (27)
**How would you rate your symptoms of DDD after having received treatment?**			
Much better/A little better	5 (29)	6 (46)	11 (37)
The same/A little worse/Much worse	5 (29)	2 (15)	7 (23)
Not applicable	3 (18)	1 (8)	4 (13)
Unanswered	4 (24)	4 (31)	8 (27)
**How satisfied were you with the treatment you have received?**			
Extremely satisfied/Somewhat satisfied	5 (29)	6 (46)	11 (37)
Neither satisfied nor dissatisfied/Somewhat dissatisfied/Extremely dissatisfied	5 (29)	3 (23)	8 (27)
Not applicable	3 (18)	0 (0)	3 (10)
Unanswered	4 (24)	4 (31)	8 (27)
**How would you rate your overall experience with this study?**			
Excellent/Very good/Good	5 (29)	7 (54)	12 (40)
Fair/Poor/Very poor	8 (47)	2 (15)	10 (33)
Unanswered	4 (24)	4 (31)	8 (27)

Participants were satisfied with the assessment process and questionnaires and how their queries were dealt with (> 50% were either extremely or somewhat satisfied).

The randomization process was not deemed acceptable, with 47% endorsing options stating they were neither satisfied nor dissatisfied or dissatisfied. Differential ratings between groups were 59% in TAU vs. 31% in CBT-f-DDD.

Ratings of overall experience of the study did not meet the acceptable range (i.e., > 50% positive responses), with 40% rating their experience as excellent, very good, or good, and 33% rating it as fair, poor, or very poor. There were differential ratings between the groups, with the TAU group rating their overall experience more negatively than the CBT group.

Questions about the acceptability of intervention were all RAG rated between green and amber (satisfaction with therapist/treatment, outcome of treatment, improvement in DDD symptoms). In the CBT group, 54% were extremely or somewhat satisfied with the therapist they worked with. Forty-six percent rated their treatment outcome positively, with the same percentage stating that their symptoms were much or a little better since receiving treatment.

There were significant missing data (27%) about acceptability, and a future study would need to improve this data collection.

#### Therapist acceptability

Thirty-six clinicians completed a feedback form after attending the half-day training workshop with a Likert scale from 0 to 8, where 8 indicated excellent. The overall mean rating was 7.6 (i.e., green RAG rated). Clinicians also gave free text comments. Several commented that the presentation was clear, with a good mix of theory, case studies, and videos of people with DDD which gave them a lived-experience perspective.

## Qualitative analyses of participants and clinician acceptability

Acceptability and treatment experiences were also explored via qualitative interviews with participants who received CBT-f-DDD (*n* = 7) and clinicians (*n* = 7), once all therapy and assessments had been completed.

### Participant analyses

CBT-f-DDD was generally well received by participants. In the context of some aversive past help-seeking experiences, participants appreciated receiving treatment specific to DDD. Nearly all reported experiencing improvements in symptoms. However, two participants viewed the therapy as useful overall, although they found it less helpful as they were unsure about their DDD diagnosis. Some described viewing DDD as more controllable and malleable than before therapy.

The key therapeutic components that participants cited as beneficial included having a strong therapeutic rapport, building a shared and informed understanding of DDD, and learning to reframe thoughts. A few participants, however, felt slightly distant from their clinicians (which may have been due to virtual sessions), and some had irregular treatment sessions, which impacted momentum and engagement. The start and end of therapy were experienced as disorganized by some participants; for example that ending treatment felt abrupt, with others adding that the onward plan after CBT-f-DDD was unclear.

### Clinician analyses

All clinicians who delivered CBT-f-CBT were interviewed. Nearly all were aged between 30 and 49 years old and female. Five clinicians were White; two were Black. Among the seven clinicians, four had clients who had completed therapy and three had clients that dropped out between sessions 2 and 4. Clinicians felt there was a clear need to increase DDD treatment provision and construct effective treatment pathways within the NHS. They also recommended that CBT-f-DD be included in training so that clinicians had the skills to offer these clients. Themes from the qualitative analysis and illustrative quotes are presented in Table 7.

**Table 7:** List of qualitative themes and quotes

Themes	Illustrative quotes
Reception of therapy	“I’m very thankful that I could be a part of this … there are too many people like me who are struggling. And we are, like, normal, functioning people … it’s, like, this horrible thing (DDD) and no one will ever tell you what’s going on with you. So, I think it’s a really important job you’re doing because you can change a lot of lives.” (Client 012) “It feels much more manageable. And I now know it’s (dissociation) not permanent … it’s in my control, and that it will go away … and it’s not scary anymore, because I know that it can change.” (Client 009)
Key therapeutic processes as drivers for change	“There was a point where we’d identified all the things that had … perpetuated my symptoms … a bunch of stuff that I’d kind of been leaning on to just get through life for a long time … I felt like that was easier to then move on and actually try all the new stuff (therapeutic techniques) … that was quite powerful.” (Client 005) “Their experience of my DDD was sort of a job and my experience of my DDD is sort of a pretty disastrous, horrendous experience … that's no bad thing they’re doing- This is their vocation. But I could certainly tell that I was one of a number that they saw in a week.” (Client 025)
Improving the therapy	“The momentum is really, really important. There were a few periods of time … I couldn’t attend therapy for a few weeks, and they were away … it felt like we were just spending sessions playing catch-up.” (Client 005) “At the end, perhaps being clearer on what if anything, happens next … Is it like, I’m discharged, and if something happened in future where I felt like I needed more help, I would start from scratch on the whole process?” (Client 001)
Treatment implementation in the NHS	“I think it seems perfectly feasible. As with any provision it has to be thought about what’s primary care, what’s secondary care, what’s the pathway … how many people are we going to get through the door … What if we (Talking Therapies) start getting people with more severe- I’m not really sure there’ll be anywhere we could send them.” (Clinician 13) “There’s so much staff turnover (in Talking Therapies services), you can’t guarantee that if you did a training in every borough, you’re going to capture everyone. Whereas I think within those courses (core CBT training), you can reach a wider net.” (Clinician 1)

#### Objective 7: Preliminary Evaluation of Participant Response to Intervention

In the CBT-f-DDD group, a larger proportion (46%, *n* = 6) said that they felt better after intervention in comparison to the TAU group (16%, *n* = 2). There was encouraging evidence of a difference in the change scores between baseline and final values of those who had been randomized to CBT versus those randomized to TAU. Those in the CBT arm had a mean decrease in CDS scores of 16.88 points (SD = 43.57) versus a mean decrease in CDS scores of 5.5 points (SD = 24.96) for those assigned to the TAU arm.

#### Statistical parameters

The correlation between outcome and baseline values for the primary outcome (CDS) was 0.847. The standard deviation of the CDS score at baseline was 55.22.

#### Objective 8: Health economic analysis

The health economic analysis estimated the costs and the Quality-Adjusted Life Years (QALYs) associated with both the intervention and the control. The cost of CBT-f-DDD and TAU was assessed taking into account the time required to administer the intervention, using the salary of the professional figures involved in delivering each session. Estimated costs were relatively similar, with CBT-f-DDD costing £90 more compared to TAU. NHS and private costs were assessed using CSRI data and unit costs publicly available (e.g., NHS tariffs) or provided by patients (average daily salary). From an NHS perspective, the cost of healthcare resources used in the CBT-f-DDD group was £207 per patient more expensive than TAU (£1647 versus £1440). When adding societal costs (private consultations, out-of-pocket medications, and productivity losses), CBT was found to save £153 per person.

EQ5D questionnaires were administered at T0, T2, and T3 to assess patient Quality of Life. Profiles were then translated into utility value using UK values, and a linear approximation method was used to assess QALYs at one year, assuming that the change in quality of life between timepoints is linear. The intervention group reported an additional 0.08 QALY per patient on average.

Adopting the NHS perspective, the Incremental Cost Effectiveness Ratio (ratio between the difference in costs and the difference in QALYs of CBT-f-DDD vs TAU), is £2590 per QALY. This is far below the recommended £20,000 per QALY suggested by NICE; therefore, CBT-f-DDD should be recommended in the NHS. When adding societal costs, the results are even more promising, because not only is CBT-f-DDD more effective, but it also saves £1919 for each extra QALY gained. A summary of costs and QALYs at 1 year in each arm is reported in Table 8.

**Table 8:** Health economic analyses: costs and outcomes at 1 year

	**TAU (*N* = 9)**	**CBT (*N* = 9)**
Cost of productivity loss	£10,290.40 (95% C.I, 9135–11,445)	£4603.60 (95% C.I, 3106–6099)
Cost to NHS	£24,489.71 (95% C.I, 18,870–30,107)	£21,414.71 (95% C.I, 19,080–23,747)
Average NHS costs	£1440.57 (95% C.I, 815–2064)	£1647.29 (95% C.I,1387–1906)
Cost of private healthcare	£1000.50 (95% C.I, 370–1630)	£2039.50 (95% C.I, 1746–2331)
Societal costs (NHS + private)	£11,290.90 (95% C.I, 10,134–12,445)	£6643.10 (95% C.I, 5146–8139)
Average societal costs	£664.17 (95% C.I, 499–829)	£511.01 (95% C.I, 137–885)
Total QALYs 1 year	5.63 (95% C.I., 5.48–5.78)	6.35 (95% C.I., 6.30–6.39)
Average QALYs 1 year	0.62 (95% C.I., 0.47–0.77)	0.70 (95% C.I., 0.65–0.74)
Total QALYs 6 months	4.17 (95% C.I., 4.06–4.3)	6.42 (95% C.I.,6.38–6.50)
Average QALYs 6 months	0.49 (95% C.I., 0.38–0.6)	0.52 (95% C.I., 0.48–0.56)

#### Harms

There were no reported incidents of harm or unintended effects in either arm of the study.

## Discussion

This study is the first to examine the feasibility and acceptability of a CBT intervention for DDD within general NHS adult mental health services. Given we are not aware of any randomized controlled trial evidence for talking therapy treatments for DDD, and no specific NICE guidance for DDD, this study represents a vital first step in the process to establish this evidence base by building on the promising results from two clinical audits into CBT-f-DDD in a specialist service. The results for the CBT-f-DDD intervention from this study are encouraging, and given that the feasibility targets were mostly met and that practical changes can be implemented to improve a future study, these results are overall supportive of a subsequent study with a larger sample size.

## Limitations

This feasibility study was valuable in highlighting several limitations which would need to be addressed in a future efficacy trial of CBT-f-DDD. These included slow recruitment and low retention rates; the need for a spectrum of severity and duration of DDD to be more representative; and changes to the design of the study to improve participants’ acceptability ratings. Even within the limited scope of a feasibility study, the overall sample size was smaller than hoped for due to recruitment and retention problems. Each of these issues with proposed amendments will be covered in each of the sections below.

### Recruitment

The study was able to recruit people with DDD, which demonstrated that there are people with a clinical need for treatment for this condition in NHS services, particularly within psychological therapy services. This is an important finding given that currently NHS primary care talking therapy services do not include routine assessment of DDD, nor do they have specific coding for those who are identified with DDD, meaning that this data is not captured. This helps to explain why people with DDD often struggle to have their DDD symptoms recognized have difficulties accessing appropriate interventions and why health services underdiagnose DDD compared to prevalence rates from survey data. The findings from this study indicate that encouraging talking therapy services nationwide to include the two diagnostic questions for DDD used in this study within routine assessments would help remedy these issues. Identifying and treating DDD at this early stage of help seeking would potentially reduce the duration of symptoms for people with DDD, as well as the health and societal burden of this condition.

Recruitment was steady, albeit slow, with only 2.3 participants recruited per month. A future efficacy study would need to have several amendments to the design: significantly more sources of recruitment, a longer period of advertising the study before recruitment started, and a longer recruitment period. In addition, if the study employed trial therapists directly rather than worked with clinicians linked to specific Trusts this would enable suitable participants recruited from the national charity to be included.

### Retention rates

The rates of retention for assessments in the study were red RAG rated, as there was a 36% drop out across both groups. There were several possible reasons for this. There were 3 research assistants over the project duration due to career advancement and therefore a lack of continuity in those conducting assessments. Following the COVID-19 pandemic, most assessments were offered online which may have reduced engagement. The measure for the assessment of comorbidity (CIS-R) was lengthy as it required a research assistant to administer and would benefit from conversion into an online format for self-completion. In terms of therapy attrition, some of this is likely to be attributable to a more transient population in London than nationally with several participants moving out of the catchment area during the study, meaning therapy could not be continued. Again, directly employing study therapists would overcome this issue in a future study as they would be able to continue to provide therapy even if participants relocated. However, on a positive note, the dropout rates for the CBT-f-DDD group were consistent with dropout rates in the clinical services from which the participants were recruited, so it did not indicate any specific issue with the CBT-f-DDD per se. However, a future study would benefit from offering more in-person sessions rather than virtual sessions as qualitative feedback indicated this was a preference in those with DDD because screen use can exacerbate their sense of disconnectedness.

### Representativeness

Overall, the sample in this study was comparable to those in previously published studies in terms of demographics, clinical, and DDD characteristics apart from four criteria: age, duration and severity of DDD, and current comorbidity. The median age of the present sample at 27 years (SD = 5.8) was younger than the samples in the two previously published studies where the mean ages were approximately 38. This sample also had a significantly shorter median duration of DDD at 4 years than in the two tertiary samples which had means of 14–15 years, as well as a lower CDS mean score of 122, compared to 152 in the latest tertiary service audit. The current sample also had a high rate of co-morbidity with 87% having another disorder, as measured by the CIS-R, compared to the most recent audit where 78% had a comorbidity. However, this difference may be due to a greater accuracy in this study where comorbidity was assessed more formally. Many of these differences are likely to be due to the sample being recruited largely from primary care talking therapies where participants are in the early stages of the condition. A younger sample with less severity and duration might help explain lower retention rates from this sample when compared to previously published samples. A future study would benefit from recruiting more participants from secondary and tertiary care services to ensure the full spectrum of DDD is included. Moreover, a slight under-representativeness in ethnic diversity was seen in this study as in the two previous audits. A future study will need to investigate how to increase recruitment from minority ethnic groups within NHS Trusts.

### Resources

We estimate that the costs of a larger fully powered study would be feasible and in line with other similar studies, especially if recruitment and retention rates are improved.

### Participant acceptability: processes, measures, and intervention

Although the end of treatment satisfaction questionnaire had no red RAG ratings and several items were green RAG rated, there were some aspects of the study where participant satisfaction was relatively low and would need improvement in a future study. There were significant amounts of missing data from the satisfaction questionnaire, and any future studies would want to address this such as by using online completion formats for questionnaires or having this completed with a research assistant, rather than self-directed.

It is unclear why the item “information about the study” was rated as only moderately acceptable as all participants were given a detailed information sheet using a standard format and had the opportunity to speak to a member of the research team before consenting. However, it is possible there was miscommunication at an earlier stage as the procedural protocol was that NHS clinical staff made the first approach to potential participants to get consent for the research team to follow up. Information about this study was largely disseminated to clinicians via their team leaders; however, in a future study, perhaps more direct communication between the study team and clinicians might be beneficial to reduce possible misinformation.

Lack of satisfaction with the randomization process was, as might be expected, mainly within the TAU group. Improving this could be achieved by offering the TAU group CBT-f-DDD therapy or a comparable treatment at the end of their participation in the study, if required, to remove the perception that they had received an inferior experience than those participants who had been randomized to the active treatment arm. However, this may be difficult in terms of requiring extra funding due to a longer trial duration and additional resources. Ratings of participant satisfaction with their therapist were much lower than expected. In addition, there was some criticism of therapy delivery such as a lack of continuity of sessions, feeling distant from their therapist, and the amount of sessions that were delivered online. Changes need to be made to a future study to address these concerns. Many of these issues were attributable to the study design, specifically using therapists within existing NHS services rather than the standard therapy trial methodology of employing trial therapists. Without having choice in the clinicians who took part in the study, there was greater variability in the expertise of the clinicians who put themselves forward to take part in the trial than ideally would be the case. Employing trial therapists directly would allow the selection of clinicians to ensure greater homogeneity of skills and experience, and therefore the intervention being delivered. Moreover, having trial therapists is likely to promote greater engagement on the part of the therapists and give them more protection from other competing demands within busy NHS services. A larger scale efficacy study could also allow therapists to have a practice client before taking on a trial client. Moreover, although clinicians rated the CBT-f-DDD training highly, a longer training period of at least 1 day would be advised to allow more time for practicing interventions to ensure greater consistency in clinician skills.

Satisfaction ratings for overall therapy outcome, symptoms after treatment, and treatment received were between amber and green RAG ratings. Although these were higher in the CBT than TAU group (46% rated these items within the good to excellent category), these ratings were still lower than would be hoped for. In discussion with people who had lived experience of DDD during a PAG meeting of the results, it was suggested that the pervasive lack of interventions for DDD could have resulted in either a hopelessness that nothing could help, or conversely for overly optimistic expectations of treatment, which if not met, had led to disappointment. Although research assistants emphasized that this was exploratory research, it is understandable that participants might have had initially high hopes for improvements with a specifically targeted intervention, although with more modest outcomes. Lastly, given this study was working within existing services and clinical pathways which did not standardly accommodate people with DDD, a future study would need to plan for what would be offered to participants who needed further support after the trial.

However, in those interviewed who had received the intervention, CBT-f-DDD was generally well-received and nearly all reported improvements in symptoms. An important finding from the qualitative interviews was that even if DDD symptom severity had not changed significantly, participants reported a change in their relationship with residual symptoms, being less afraid and more able to live with symptoms, as well as feeling their DDD had become more controllable. This was confirmed in the economic analysis which found that CBT-f-DDD not only improved quality of life but from a societal perspective could also save money in terms of avoided productivity losses. Measures to assess these other aspects of change in addition to DDD symptom reduction should be included in any further studies.

Therapist ratings of acceptability were overall good. The training and supervision provided clinicians with a protocol to assess, formulate and treat DDD within the context of comorbid conditions, with many reporting an increase in their knowledge, skills and confidence in working with this client group. Clinicians felt it was important that clinical services and pathways should be developed and specific teaching on DDD be included in standard CBT training courses.

### Preliminary evaluation of participants’ response to intervention

Given that this is a feasibility study, we could not conduct inferential analyses as the study is highly likely to have been underpowered. The current consensus on the appropriate statistical analyses in feasibility studies is summarized: “… most pilot studies should not be used to estimate effect sizes, provide power calculations for statistical tests or perform exploratory analyses of efficacy” [24].

The sample size for this study is determined by guidelines on the size of feasibility studies; we did not power the study on a Minimum Clinically Important Difference (MCID). The literature supports a sample size of approximately 30 participants as being sufficient to establish the feasibility of a larger study [25].Therefore, the standard errors and 95% confidence intervals around the estimate of the primary outcome do not provide helpful data on the estimation of the sample size for either this feasibility study, or for the subsequent fully powered study we intend to carry out. The standard deviation of the CDS score at baseline was large (55.22). The implications of this are that the intervention may work very well for some people and not for others, so future research may need to explore moderators of response.

We cannot comment on any differences between the groups in terms of our primary outcome. We provide an estimate and 95% confidence intervals that indicate, as expected, that a larger sample would be required for a fully powered trial. We estimate that over an 18-month recruitment period, it could be possible to recruit 40–60 per site across 5 sites covering both rural and urban areas.

### Health economics

The health economics analyses found that when considering the societal perspective, the CBT-f-DDD saves £153 per person and gains an additional 0.08 QALY per person. This is a positive finding and suggests that a larger full trial would be recommended in the NHS.

### Feasibility results indicating progression to full trial

The results of the a priori progression criteria that needed to be met to establish the feasibility of the full trial (Table 2) indicated that there were 6 items green RAG rated (therapist attrition, overall resources, participant acceptability for the assessment process, questionnaires and queries, as well as therapist acceptability). There were 8 items RAG rated between green and amber (therapist adherence, participant acceptability for study information, randomization, therapist, outcome of treatment, symptoms of DDD post intervention, treatment satisfaction, and overall study experience). Two items were red RAG rated (participant recruitment and retention). There are several suggested improvements that are needed to refine the current study. These include changes to improve recruitment and retention, as well as alterations to trial design and procedures to aid optimization of intervention delivery. Once these are implemented, we believe a future, larger, adequately powered trial is indicated.

## Conclusion

The current study found that within NHS talking therapy services clinicians were able to identify people with DDD and were able to treat these symptoms to a generally acceptable level of patient satisfaction. This feasibility study has been valuable in highlighting specific changes such as recruitment, retention and refinements to the trial design that would be needed for a larger, adequately powered efficacy trial. If such further studies can establish the effectiveness of CBT-f-DDD and the treatment is rolled out within NHS services, we hope that by delivering this treatment to people with DDD we can reduce the burden of illness on the individual as well as having wider societal benefits by saving money from service usage and increased productivity in the longer term.

## Data Availability

The datasets generated and analyzed during the current study are not publicly available given these data could have the potential for reverse deanonymization of participants. However, these are available from the corresponding author on reasonable request.
